# Influence of the parameters of the convolutional neural network model in predicting the effective compressive modulus of porous structure

**DOI:** 10.3389/fbioe.2022.985688

**Published:** 2022-09-15

**Authors:** Yongtao Lu, Yi Huo, Zhuoyue Yang, Yibiao Niu, Ming Zhao, Sergei Bosiakov, Lei Li

**Affiliations:** ^1^ Department of Engineering Mechanics, Dalian University of Technology, Dalian, China; ^2^ DUT-BSU Joint Institute, Dalian University of Technology, Dalian, China; ^3^ Xi’an Aerospace Propulsion Institute, Xi’an, China; ^4^ Faculty of Mechanics and Mathematics, Belarusian State University, Minsk, Belarus; ^5^ Department of Vascular Surgery, The Second Affiliated Hospital of Dalian Medical University, Dalian, Liaoning, China

**Keywords:** convolutional neural network, bone scaffold, finite element modeling, sensitivity analysis, compressive modulus

## Abstract

In recent years, the convolutional neural network (CNN) technique has emerged as an efficient new method for designing porous structure, but a CNN model generally contains a large number of parameters, each of which could influence the predictive ability of the CNN model. Furthermore, there is no consensus on the setting of each parameter in the CNN model. Therefore, the present study aimed to investigate the sensitivity of the parameters in the CNN model for the prediction of the mechanical property of porous structures. 10,500 samples of porous structure were randomly generated, and their effective compressive moduli obtained from finite element analysis were used as the ground truths to construct and train a CNN model. 8,000 of the samples were used to train the CNN model, 2000 samples were used for the cross-validation of the CNN model and the remaining 500 new structures, which did not participate in the CNN training process, were used to test the predictive power of the CNN model. The sensitivity of the number of convolutional layers, the number of convolution kernels, the number of pooling layers, the number of fully connected layers and the optimizer in the CNN model were then investigated. The results showed that the optimizer has the largest influence on the training speed, while the fully connected layer has the least impact on the training speed. Additionally, the pooling layer has the largest impact on the predictive ability while the optimizer has the least impact on the predictive ability. In conclusion, the parameters of the CNN model play an important role in the performance of the CNN model and the parameter sensitivity analysis can help optimize the CNN model to increase the computational efficiency.

## Introduction

In recent years, the machine learning and deep learning techniques have emerged as new techniques widely used in many fields, including in the prediction of the mechanical properties of materials and the design of porous structures.

In the field of prediction of the mechanical properties of materials, Li et al. (2019) used the machine learning method to establish the implicit mapping between the effective mechanical property and the mesoscale structure of heterogeneous materials, and the results showed that the machine training model can accurately predict the elastic modulus of the structures. However, this study simplified the parameters in the CNN model through algebraic operations and the influence of parameters on the CNN model is not mentioned. Wu et al. (2019) proposed a machine learning method for predicting the effective diffusivity of two-dimensional porous structures, and the results showed that this model can accurately predict the transport properties of porous structures. Besides, one of the key issues of the research is how to select the parameters to avoid overfitting the CNN model. Graczyk and Matyka (2020) utilized CNN to predict porosity, permeability and tortuosity of porous structures, and found that the error between the predicted results of the CNN model and the theoretical value can be kept in a very small range. Besides, the parameter is reduced by 10% every 50 epochs of the training. Paul and Bhattacharjee (2018) used artificial neural network (ANN) to investigate the effects of temperature, humidity and other conditions on the properties of metal materials, and the accuracy of the ANN network prediction of fracture toughness of metal materials is close to 80%. Besides, machine learning is also widely used in some biological problems of bone tissue replacement. For example, Wu et al. (2021) proposed a new model based on machine learning techniques to predict bone ingrowth. Compared with the traditional FE model, the new prediction model of bone ingrowth based on machine learning has higher efficiency and accuracy.

In the field of the design of porous structures, Gu et al. (2018) proposed a method to design hierarchical materials using machine learning, and the results showed that tougher and stronger materials can be obtained by the method. It is worth pointing out that the study built blocks into a single unit cell to reduce the number of parameters needed in their machine learning model. Tan et al. (2020) proposed a deep learning model based on CNN for the design of microstructural materials, and found that the obtained microstructural materials with desired compliance tensor showed better mechanical properties. Besides, the proposed model reduced the number of design parameters from 4,096 to 25. Wang et al. (2018) reconstructed porous structures using the CNN model, and the results showed that the proposed method greatly improved the connectivity of the structures and exhibits better performance than conventional methods. Bessa et al. (2017) developed a new data-driven computing framework to assist in structural design, using machine learning to replace empirical constitutive models with experimental data. The results show that the mean square errors, mean relative errors, and the absolute fraction of variance values of the ANN for predicting friction factors were 1.26, 4.36 × 10^−7^ and 0.9993, respectively. Besides, the bone scaffold is used to replace the defected bone tissues and the machine learning has been widely used in the design of bone scaffolds, Conev et al. (2020) trained a machine learning model to predict the printing quality for a given printing configuration accurately for the material. They identified which parameters mostly affect the printing quality. Thus, the proper bone scaffolds can be designed to avoid poor print quality. Cilla et al. (2017) used machine learning techniques to optimize the bone joint replacements, which can be used in the field of the design of the bone scaffolds.

These previous studies showed that machine learning and deep learning techniques are of great significance in the fields of the prediction of mechanical properties of materials, the design of porous structures and so on. Besides, the previous study also pointed out the importance of parameter sensitivity. However, the parameter sensitivity studied in these models has not been fully understood. Therefore, it is of great importance to study the parameter sensitivity of CNN model and improve the efficiency of machine learning.

The CNN is one main branch of the deep learning techniques and the CNN networks are inspired by biological processes, in which the connectivity pattern between neurons resembles the organization and each neuron in one layer are connected to all neurons in the next layer. The CNN models consist of convolutional layers, pooling layers, and fully connected layers (Yamashita et al., 2018). The parameters of each layer of the neural network have a certain influence on the training speed and prediction ability of the entire convolutional neural network. Thus, finding the optimal parameter design is one of the important parts of the training of the convolutional neural network. However, the convolutional neural network needs to be re-trained when the parameters are adjusted. Therefore, using the optimal parameters plays a crucial role in saving computational time and increasing the model performance. However, the parameter sensitivity analysis has not been fully understood in the previous studies, which means that the prediction performance of the CNN models may be further improved and the computational efficiency can be further improved.

The present study aimed to investigate the sensitivity of the parameters in the CNN model for the prediction of the mechanical property of porous structures. A CNN model with convolutional layers, pooling layers and fully connected layers was first constructed to predict the effective compressive modulus of porous structures and then the influence of various parameters in the CNN on the training speed and prediction ability of the CNN model was investigated.

## Materials and methods

### The design setting of the 2D porous structure and the CNN model

**FIGURE 1:**
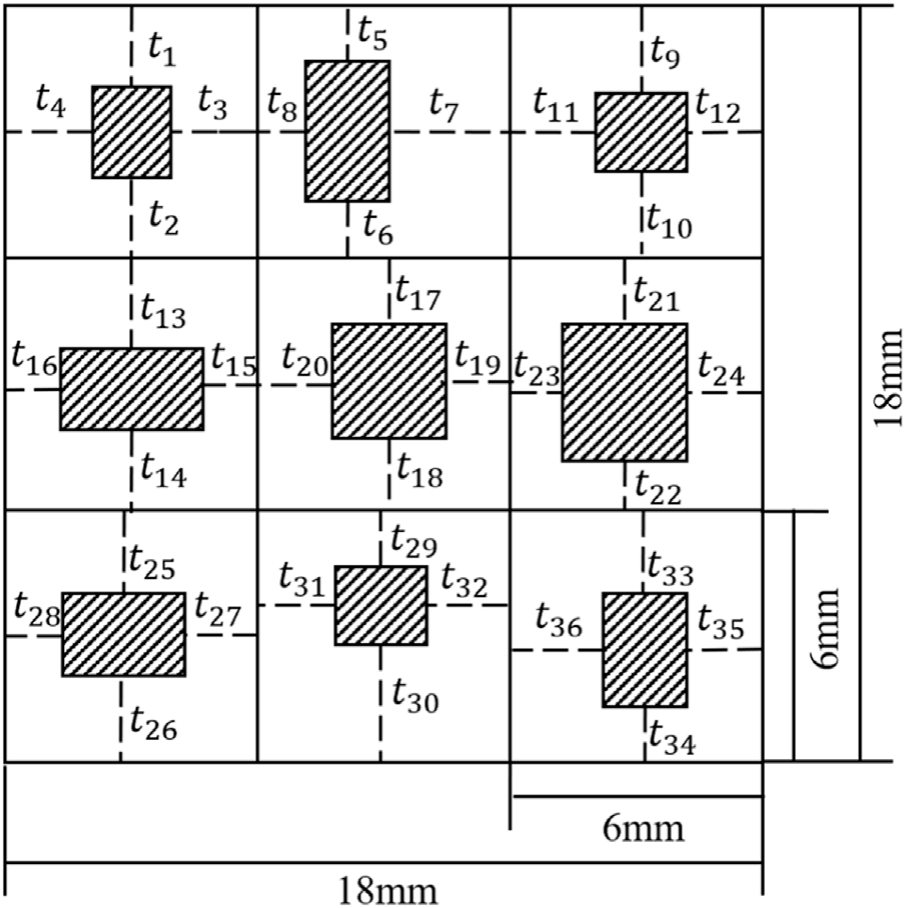
Schematic diagram of porous composite structure (The shaded areas are the pores).

In the present study, a two-dimensional (2D) porous structure was used as an example to investigate the parameter sensitivity of a convolutional neural network in predicting the mechanical property of the porous structure. Because the CNN model needs too many parameters, it is unrealistic to study all the parameters one by one, so 2D models are used in most of the current studies. For example, Wu et al. (2019) used 2D porous structures to simplify the parameters and it has been proved that the accuracy of the 2D model can meet the requirements. The 2D 3 × 3 porous structure was used for the demonstration in the present study (Figure 1). The dimension of the structure was set to 18.0 × 18.0 mm. In each cell of the structure, the four-dimensional parameters were set as the independent design variables. Therefore, for the entire structure, there are 36 independent design variables $\left(t_{1}∼t_{36}\right)$ . It is worth noting that the emergence of additive manufacturing (AM) enables the fabrication of structures with complex geometries, especially the porous structures. For example, Gu et al. (2018) used AM technique to manufacture and test the enhanced porous structures. Since in the previous studies, the fabrication of porous structures was achieved by AM technique, it is meaningful to consider the constraints of AM technique. The additive manufacturing constraint puts additional requirements for the dimension and aperture of the porous structure, i.e., the minimal thickness of the struts should be larger than the precision of the AM technique (0.2 mm in the present setting). To meet these requirements, the minimum unit for changing the dimensional variables was set to 0.2 mm. In other words, there are only three possibilities for $t_{1}∼t_{36}$ , i.e., 0.2 mm, 0.4 mm and 0.6 mm.

**FIGURE 2:**
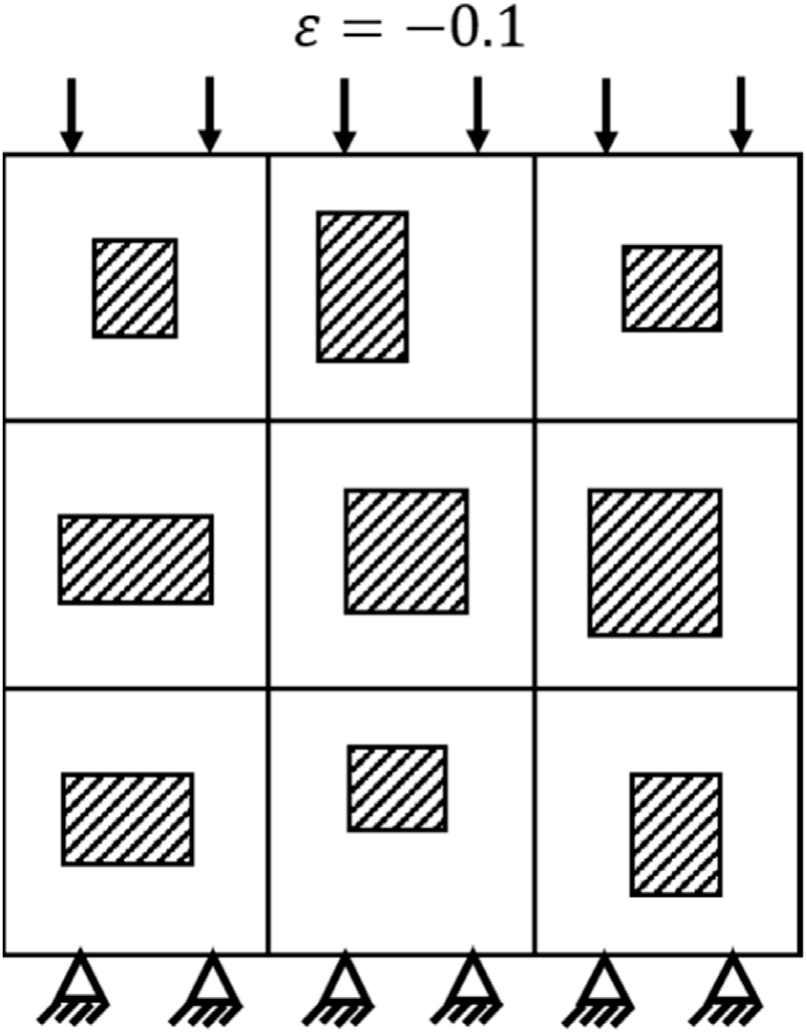
Loading and constraint defined in the finite element analysis (“ε” refers to the strain).

The effective compressive moduli of the porous structures calculated from the finite element (FE) analysis were taken as the ground truths and used to train the CNN model constructed in the present study. As shown in Figure 2, the nodes on the one (down) side of the porous structures were fully constrained while the nodes on the opposite (up) side were subjected to a compressive displacement loading. The effective compressive modulus of the porous structures was calculated by the total reaction force and the displacement applied. The Ti-6Al-4V was used to make the porous structure. Therefore, in the FE model, the Young’s modulus of the solid part was set to 113.8 GPa and the Poisson’s ratio was set to 0.34 (Niinomi, 1998).

**FIGURE 3:**
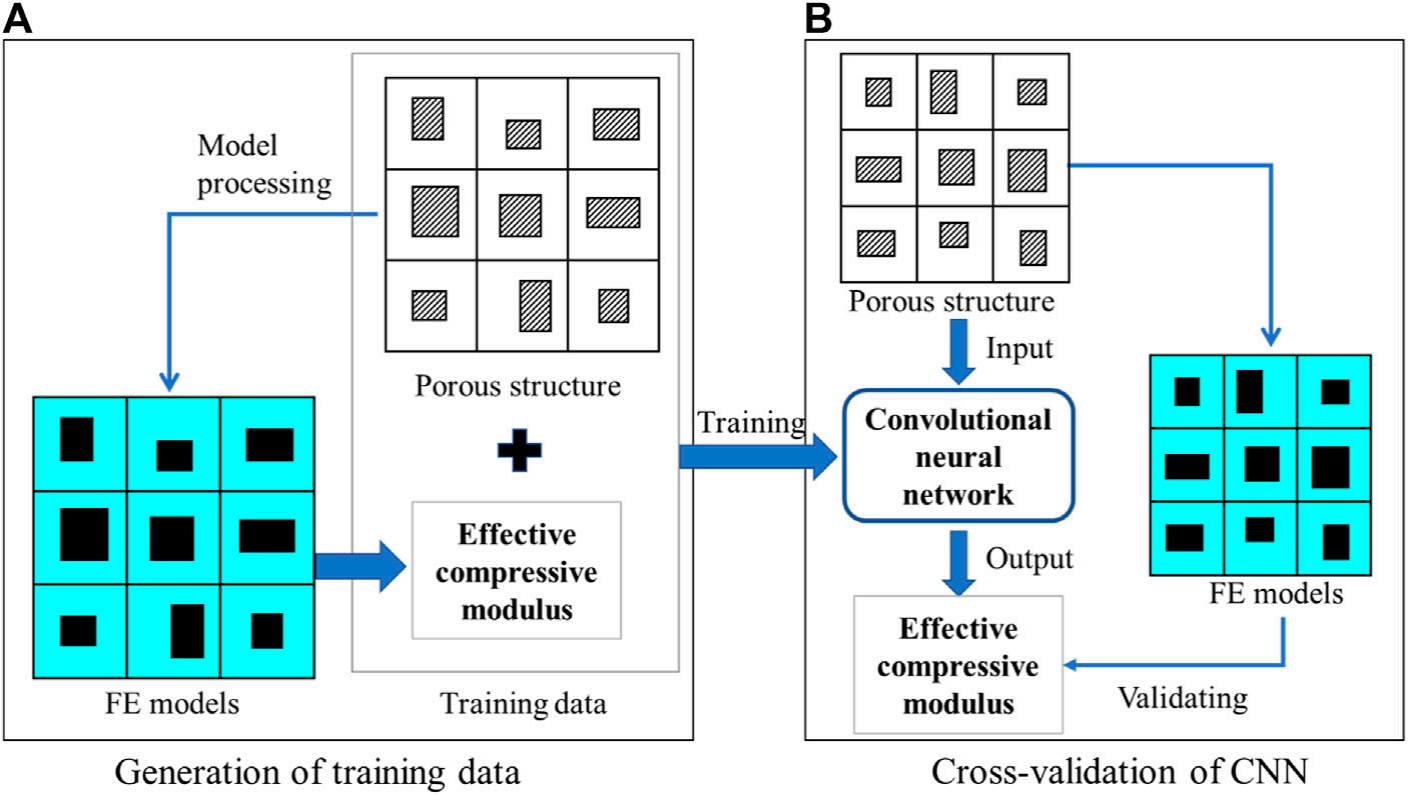
The workflow for the **(A)** training and **(B)** cross-validation of the convolutional neural network (CNN) model.

A CNN model was trained firstly and the procedure for the training and testing of the CNN model is presented in Figure 3. The training process of the CNN model can be briefly described as below: First, 10,000 groups of variables were randomly generated and each group of variables contained 36 independent variables $\left(t_{1}∼t_{36}\right)$ . Then, the corresponding 1000 FE models were built based on these dimensional variables. The effective compressive modulus of each sample was obtained from FE calculation and used as the ground truth for building the CNN model. Among the 10,000 samples, 8,000 were used for the training (Figure 3A), and 2,000 were used for the cross-validation (Figure 3B). It should be noted that both the 8,000 training samples and the 2000 cross-validation samples are involved in the adjustment of the parameters of the CNN model. The 2000 cross-validation samples have to be different from the 8,000 training samples. Otherwise, the CNN models constructed will have no ability to predict the elastic modulus of new porous structures.

**FIGURE 4:**
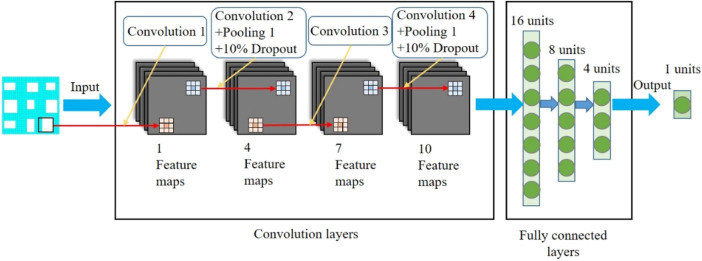
The convolutional neural network model constructed in the present study.

The constructed CNN model is shown in Figure 4 and was guided by the work done by Li et al. (2019) which was created to solve a similar problem, i.e., predicting the mechanical property of porous materials using image based deep learning technique. It should be noted that there are many different types of CNN models in the literature and the CNN model in the present study is just one demonstration of the parameter sensitivity study. In the CNN model constructed (Figure 4), the input is the design of the porous structure (Figure 1) and the output is the effective compressive modulus of the input porous structure. In the CNN model constructed, four convolutional layers, two pooling layers and three fully connected layers were applied to the image. The size of all the convolution kernels was set to 3 × 3. The maximal pooling was applied after the convolutional layers to simplify the information of the output neurons. To improve the accuracy of the CNN model, 10% dropout was used after the two pooling layers. A convolutional layer is the main building block of the CNN model, which contains a set of kernels learned throughout the training process. A pooling layer is usually incorporated between two successive convolutional layers. The pooling layer reduces the number of parameters and computational by down-sampling the representation. The fully connected layers are the last a few layers where all the inputs from the previous layer are connected to every neuron of the next layer. In the CNN model constructed in the present study, the fully connected layers compile the data extracted from the convolutional layers to form the final output. The CNN model proposed by Carneiro et al. (2017) can be seen as a typical CNN model, which contains the convolutional layers, pooling layers and fully connected layers. The convolutional layers usually contain the model parameters formed by the input weight matrices.

In the training process, because the purpose of the CNN model was to make an accurate prediction of the effective modulus of the porous structures, a loss function was defined to quantify the difference between the effective compressive modulus predicted from the CNN model and those calculated from the FE analysis, which was taken as the golden answers. Then, the kernels and biases in the convolutional layers and the weights in the fully connected layers were adjusted using the backpropagation algorithm (Rubio et al., 2011). In the present study, the mean absolute error (MAE) was set as the objective function:

$$\mathrm{MAE}\left[Y,f\left(X\right)\right]=\frac{1}{n}\sum_{i=1}^{n}\left|Y-f\left(X\right)\right|$$ (1)

where *Y* is the effective compressive modulus of the porous structures calculated from the FE analysis; f(X) is the corresponding effective compressive modulus calculated from the CNN model and *n* is the number of samples used for the cross-validation (*n* = 2000 in the present study).

The CNN model was built using the Tensorflow 2.0 module in Python 3.7. The training process was conducted on a desktop computer with the setting of i7-8700 CPU, 32G RAM, and the Nvidia GTX1060. The batch size was set to 128 and the training was iterated for 50 epochs.

### Parameter sensitivity analysis

The CNN model mainly consisted of convolutional layers, pooling layers, and fully connected layers, among which the pooling layers and dropout were interspersed. In the present study, the parameter sensitivity analysis was carried out by changing some parameters and maintaining other parameters unchanged in each analysis. The parameters involved are the number of convolution layers, the number of convolution kernels, the number of pooling layers, the number of the fully connected layer and the optimizer. Specifically, the number of convolutional layers was chosen from 4, 6 and 8 layers, the size of the convolution kernel in each convolutional layer was chosen from 2, 4, 6, 8, 10 and 12, the number of pooling layers was chosen from 0 to 1, the number of fully connected layers was chosen from 2, 3 and 4 and the optimizer was chosen from AdaGrad, RMSprop (Elyanow et al., 2020) and Adam (Christiansen et al., 2018). After the training, 500 new samples that did not participate in the training and cross-validation process were randomly generated (following the same procedure for generating the 10,000 samples) to assess the predictive ability of the CNN model.

The samples of porous structures firstly passed through the convolutional layer, then through the pooling layer, and finally through the fully connected layer. Therefore, the parameter sensitivity study was divided into four stages: 1) The sensitivity of the convolutional layer, 2) the sensitivity of the pooling layer, 3) the sensitivity of the fully connected layer and 4) the sensitivity of the optimizer. The best parameter design from the previous stage was retained when entering the next stage. In the initial design, there were 4 convolutional layers, 2 the pooling layers, 3 fully connected layers and the optimizer was RMSprop.

The parameter sensitivity was investigated firstly using the speed of convergence. In the present study, the CNN model was considered convergent when the MAE is stabilized and below 200.0 MPa. It should be noted the convergence condition depends on the applications and could be different in different scenarios. The parameter sensitivity was then investigated using the relative prediction error (RPE), which is defined as below:

$$\mathrm{RPE}=\frac{\left|P^{\mathrm{CNN}}-P^{\mathrm{RVE}}\right|}{P^{\mathrm{RVE}}}\times 100\%$$ (2)

where $P^{\mathrm{CNN}}$ is the effective compressive modulus calculated from the CNN model and $P^{\mathrm{RVE}}$ is the corresponding value calculated from the FE analysis.

## Result

### Training and cross-validation of the CNN model

**FIGURE 5:**
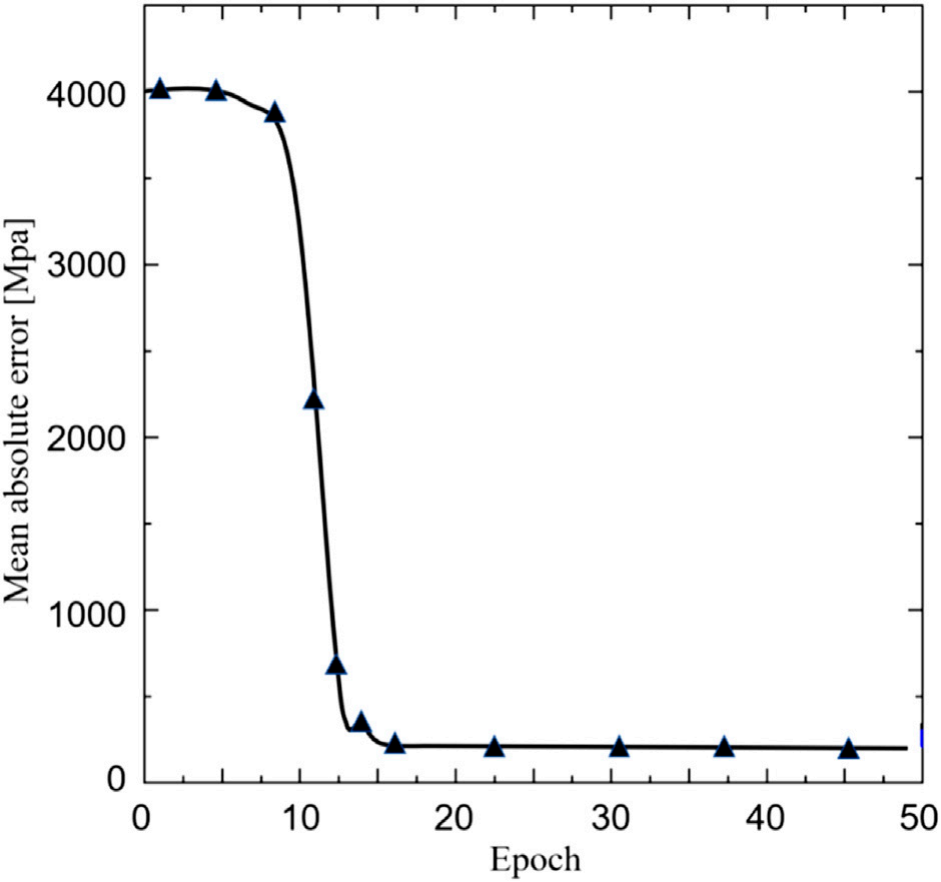
The relationship between the mean absolute error and the Epoch.

The relation between the mean absolute error (MAE) and the training iteration is shown in Figure 5. Since the initial values of the weights and biases are randomly assigned, the MAE at the first a few iterations is high. However, after several iterations, the MAE rapidly descends. Therefore, no over-fitting is observed in the cross-validation.

### Influence of parameters on the convergence speed and predictive power of CNN model

**FIGURE 6:**
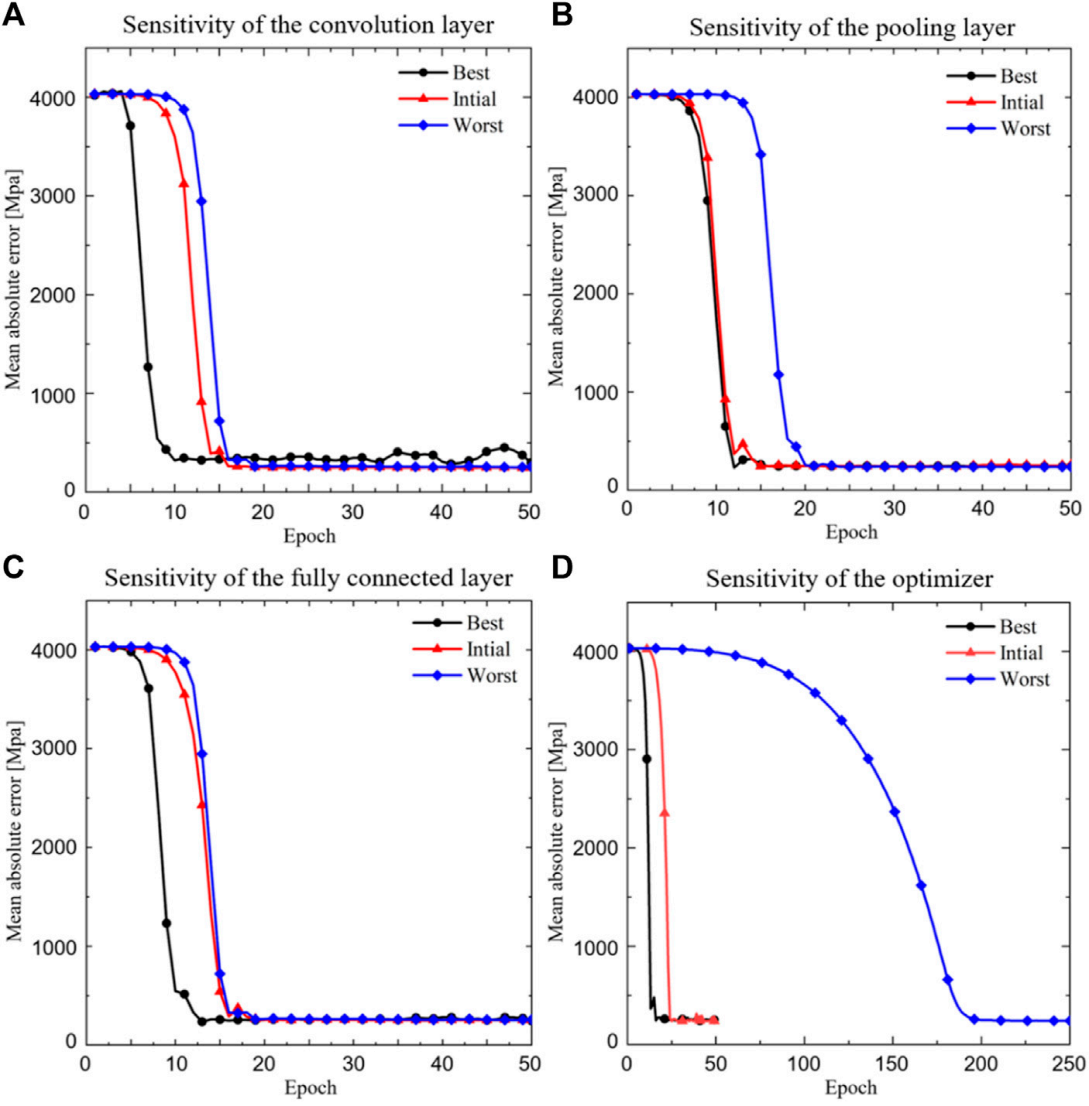
The relationship between the mean absolute error and the epoch. **(A)** Sensitivity of the convolution layer; **(B)** Sensitivity of the pooling layer; **(C)** Sensitivity of the fully connected layer and **(D)** Sensitivity of the optimizer (‘Best’ refers to the case with the fastest iterative convergence speed and the corresponding best parameters are listed in Table 1).

The influence of parameters in the CNN model on the convergence speed is shown in Figure 6, where the black line represents the fastest convergence speed, the red line represents the initial convergence speed and the blue line represents the slowest convergence speed. It is shown that when the convolutional layer is modified, the convergence of the CNN model can be changed from 10 epochs (the best scenario) to 17 epochs (the worst scenario) (Figure 6A). When the pooling layer is modified, the convergence of the CNN model can be changed from 10 epochs to 20 epochs (Figure 6B). When the fully connected layer is modified, the convergence of the CNN model can be changed from 12 epochs to 18 epochs (Figure 6C). When the optimizer is modified, the convergence of the CNN model can be changed from 10 epochs to 200 epochs (Figure 6D). The best parameters corresponding to Figures 6A–D are listed in Table 1 (a–d).

**TABLE 1:** The parameters used in the fastest convergent scenario.

	Number of convolutional layers	Number of convolutional kernels	Number of pooling layers	Number of convolutional layers	Optimizer
(a)	2	2, 4	2	3	RMSprop
(b)	2	2, 4	1	3	RMSprop
(c)	2	2, 4	1	2	RMSprop
(d)	2	2, 4	1	2	Adam

**FIGURE 7:**
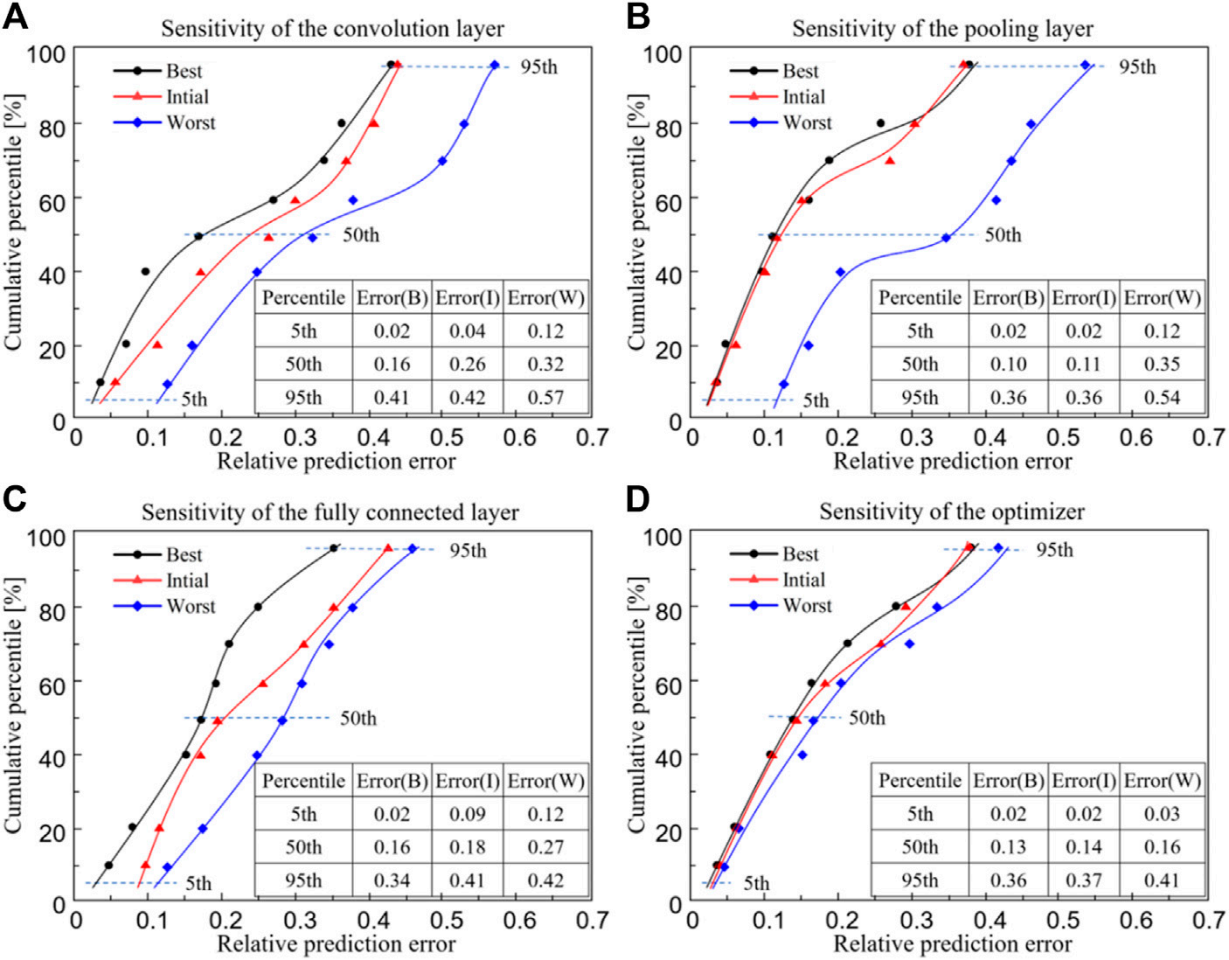
The relationship between the cumulative percentile and the relative prediction error from the convolutional neural network model. **(A)** Sensitivity of the convolution layer; **(B)** Sensitivity of the pooling layer; **(C)** Sensitivity of the fully connected layer and **(D)** Sensitivity of the optimizer (‘Best’ refers to the case with the lowest 95th percentile of relative prediction error and the corresponding best parameters are listed in Table 2).

The influence of parameters in the CNN model on the predictive power is shown in Figure 7, where the black line represents the best prediction, the red line represents the initial prediction and the blue line represents the worst prediction. Since the data are not normally distributed, the 5th, 50th and 95th percentiles are reported. Regarding the 95th percentile of the relative prediction error, it is shown in Figure 7 that when the convolutional layer is modified, the error can be changed from 0.41 (the best scenario) to 0.57 (the worst scenario) (Figure 7A); when the pooling layer is modified, the error can be changed from 0.36 to 0.54 (Figure 7B); when the fully connected layer is modified, the error can be changed from 0.34 to 0.42 (Figure 7C) and when the optimizer is modified, the error can be changed from 0.36 to 0.41 (Figure 7D). The best parameters corresponding to Figures 7A–D are listed in Table 2 (a–d).

**TABLE 2:** The parameters with the lowest 95th percentile of relative prediction error.

	Number of convolution layers	Number of convolution kernels	Number of pooling layers	Number of convolution layers	Optimizer
(a)	4	2, 4, 6, 8	2	3	RMSprop
(b)	4	2, 4, 6, 8	0	3	RMSprop
(c)	4	2, 4, 6, 8	0	2	RMSprop
(d)	4	2, 4, 6, 8	0	2	Adam

## Discussion

In the present study, a convolutional neural network (CNN) model for predicting the effective compressive modulus of the porous structures was developed and a parameter sensitivity analysis was performed to investigate the influence of parameters on the convergence speed and predictive power of the CNN model. The present study showed that the parameter sensitivity analysis is a crucial step in the development of the CNN model and can help optimize the CNN model to improve its efficiency. It should be noted that in the present study, the CNN model was chosen as one representative machine learning model to demonstrate the parameter sensitivity. Nevertheless, the conclusion made in the manuscript, i.e., it is very necessary to conduct a sensitivity analysis when a new model is constructed. Besides, the sensitivity analysis should be not only limited to the CNN model but also applicable to most machine learning and deep learning models.

In the present study, the performance of the CNN model was evaluated using both the iterative convergence speed and the relative prediction error. In terms of the convergence speed, after modifying the convolutional layer, the pooling layer, the fully connected layer and the optimizer, the differences between the best and worst numbers of iterations when CNN models are converged are 7 epochs, 10 epochs, 6 epochs and 190 epochs, respectively. It should be noted that the epochs refer to the iteration number of the CNN model. To increase the prediction accuracy of the CNN model, the CNN model will update its parameters in each iteration (epoch). Therefore, the epochs will increase the performance of the CNN model, and will indirectly affect the performance of the porous structure if the machine learning based inverse design framework is used in the design of porous structures (Wang et al., 2022). Regarding the optimizer, the results showed that the optimizer has the largest impact on the speed of the training convergence, while the fully connected layer has the least impact on the speed of the training convergence. Therefore, it is recommended to choose Adam or RMSprop instead of AdaGrad as the initial optimizer for training the CNN model in the present study. The reason why AdaGrad is slow is that Adaptive Gradient (AdaGrad) can adjust a different learning rate for each different parameter, updating important parameters with smaller steps and less important parameters with larger steps (Duchi et al., 2011). Since the CNN model constructed in the present study is a basic and simple one, the convergence speed of the AdaGrad optimizer is relatively slow. However, it is undeniable that the AdaGrad optimizer would have a good performance in other complex CNN models (Yuan et al., 2019).

Regarding the influence of the parameters in the CNN model on the predictive power of the CNN model, the results showed that the convolutional layer and pooling layer have a larger impact, while the fully connected layer has the least impact. Therefore, to improve the prediction power of the CNN model, it is preferable to adjust the pooling layer and the convolutional layer. It should be noted that the input sample used in this study is a 6×6 matrix and each feature point is equally important. Therefore, increasing the pooling layer would ignore the important feature points, which would reduce the computation time but decrease the prediction power (You et al., 2021). Consequently, the pooling layer has a great influence on the predictive ability of the CNN model in this study. For samples with insignificant feature points, adjusting the convolutional layer may have a larger influence on the predictive power of the CNN model.

It should be noted that in the present study, the ‘best’ refers to the case with the fastest iterative convergence speed in Figure 6, or the best prediction accuracy in Figure 7, and the ‘worst’ refers to the case with the slowest iterative convergence speed in Figure 6, or the worst prediction accuracy in Figure 7, and the ‘initial’ refers to the setting of 4 convolutional layers, 2 the pooling layers, 3 fully connected layers and the optimizer of RMSprop. The initial setting of the parameters is taken from Li et al. (2019). It should be noted that it is hard to find a set of parameters that make the CNN model possess the fastest convergence speed and the best prediction accuracy at the same time. Therefore, depending on the specific requirements, the definition of ‘best’ would be different in different scenarios. In the present study, the convergence speed and the prediction accuracy were set as two independent parameters in the parameter sensitivity analysis, and consequently the parameters which influence the convergence speed and the prediction accuracy were found respectively. On the other hand, it should be noted that there are two types of parameters in the CNN model. The first is the design parameters, such as the number of convolutional layers and the pooling layers, etc. These parameters are adjusted one by one and while one parameter is changing, other parameters are maintained until the expected convergence was achieved. The second is the internal parameters of the CNN model, which are adjusted internally and automatically in the training process of the CNN model. In the present study, the Tensorflow is used and thus the changing of the internal parameters cannot be visualized, but they are adjusted by the program itself to increase the predictive ability of the CNN model.

It is shown in the present study that different parameters have different influence s on the performance of the CNN model and thus it is necessary to perform the parameter sensitivity analysis when a new CNN model is constructed and additionally the parameter sensitivity analysis can help optimize the CNN model to improve the computation efficiency. In some previous studies, the sensitivity analysis of the parameters of the sample is performed using the CNN model, but the sensitivity of the parameters of the CNN model itself is always ignored. Furthermore, it should be noted that the parameter sensitivity study of the newly constructed CNN model is always skipped in the previous studies. For example, Xiao et al. (2021) developed a CNN model to evaluate the anisotropic elastic behaviors of trabecular bone. In the study, four different dual energy x-ray absorptiometry (DXA) projections (i.e., one, three, six and nine) and seven DXA image resolutions (i.e., 0.05 mm/pixel, 0.15 mm/pixel, 0.3 mm/pixel, 0.6 mm/pixel, 1.2 mm/pixel, 2.0 mm/pixel and 3.0 mm/pixel) are used to train the CNN model. It should be noted that if the sensitivity analysis of the parameters of CNN was carried out, the prediction power of the CNN model could be further improved. Although the parameter sensitivity analysis of the CNN model is performed in some studies, it is not comprehensive. For example, to predict the mechanical properties of various soils, Ng et al. (2019) identified the important spectral wavelengths using the CNN model. In their study, the sensitivity analysis of only the convolutional layer was investigated and the sensitivity analysis showed that a high prediction power with *R* ^2^ = 0.95 can be achieved using a suitable convolutional layer. It can be seen from their study that the sensitivity analysis can help select the parameters that increase the prediction power of the CNN model. However, only the sensitivity of one parameter was investigated in their study. In comparison, in the present study, the sensitivity of most parameters in the CNN model was analyzed.

Some shortcomings in the present study should be noted. First, although the sensitivity of the parameters in the CNN model was investigated in the present study, it still did not consider all the parameters. For example, the size of padding in the convolutional layer (Khanolkar et al., 2021), the choice of the activation function (Leshno et al., 1993), the size of dropout (Srivastava et al., 2014) and other parameters are not considered. There are still many parameters in the CNN model the sensitivity of which still needs to be analyzed. In addition, the sensitivity of the parameters is also different for different input samples. Nevertheless, these do not compromise the message delivered in the present study, i.e., a parameter sensitivity analysis is required to optimize the CNN model when a new model is built. Second, there is a lack of real data support for the experiment to validate the prediction. Nevertheless, the FE modeling technique has been widely accepted as a reliable technique for predicting the effective compressive modulus of porous structure. For example, de Galarreta et al. (2020) performed FE analysis of porous structures to investigate the modulus and yield strength. Besides, the compression test was performed to verify the modulus and yield strength of the structures. The results showed that the FE simulation results were in good agreement with the experimental results, which means FE modeling technique is reliable. Despite this, it is still worthy to validate the predictions of the CNN models, especially for the post-elastic mechanical properties of the porous structure, such as ultimate strength. Third, when using different samples to train the CNN model, the influence of the parameters in the CNN model on the convergence speed and prediction power may be different. Therefore, the results of the present study may not be directly used when training a new CNN model and a new parameter sensitivity analysis is required. Nevertheless, the present study showed that it is crucial to perform a parameter sensitivity analysis when a new CNN model is built, which is always ignored or skipped in the previous studies. Last but not least, 2D analysis instead of 3D was performed in the present study. It should be noted even in the 2D analysis, a large computational complexity was involved, i.e., 10,500 samples and 36 independent design variables in each sample were involved. In the setting of the present study, the number of independent design variables will be exponentially increased for the 3D case, which will create a computational “disaster” in the machine learning study. On the other hand, the 2D-based analysis has played an important role in some applications, such as in the prediction of bone strength using the 2D DXA-based images (Lu et al., 2019). Therefore, despite the 2D analysis being performed, the aim of the present study has been successfully demonstrated, i.e., the importance of parameter sensitivity analysis in the CNN model development and model optimization. On the other hand, in the 3D application, the issues with biocompatible performance of the scaffolds should also be dealt with (Shuai et al., 2020).

## Conclusion

In conclusion, it is revealed in the present study that the parameters in the CNN model have a big influence on both the convergence speed and the predictive power. Therefore, it is very necessary to conduct a sensitivity analysis when a new CNN model is developed. Additionally, the parameter sensitivity analysis can help effectively reduce the training time of the CNN model and improve the prediction power of the CNN model. From the parameter analysis, it is theoretically possible to construct the most suitable CNN model which possesses a fast convergence speed and a high prediction power.

## Data Availability

The original contributions presented in the study are included in the article/Supplementary Material, further inquiries can be directed to the corresponding author.
